# Test of the minimum supero-inferior femoral neck diameter as a sex predictor in a contemporary documented osteological collection from Portugal

**DOI:** 10.1093/fsr/owae045

**Published:** 2024-08-05

**Authors:** Leandro H Luna

**Affiliations:** CONICET, University of Buenos Aires, Faculty of Odontology, Endodontics Chair and Public Health Research Institute (IISAP), Bioarchaeology and Forensic Anthropology Research Unit (UIBAF), Buenos Aires, Argentina

**Keywords:** forensic sciences, forensic anthropology, sexual dimorphism, postcranial metrics, documented collection

## Abstract

Adult sex estimation is one of the first and most important steps in forensic examination. While dealing with disturbed burials, the most dimorphic anatomic areas of the skeleton (such as the coxae, the skull and the head of femur and humerus) may be deteriorated or fragmented. In contrast, the minimum supero-inferior femoral neck diameter (SID) is generally much better preserved. The aim of the present research is to identify the discriminatory potential of SID for sex estimation and to test different formulae and mathematical procedures currently available in the forensic literature, on a sample of 295 contemporary individuals from the 21st Century Identified Skeletal Collection (University of Coimbra, Portugal), in order to identify its relevance for application in Portuguese forensic cases. Results showed that SID is a dimorphic variable, with high frequencies and probabilities of cases correctly estimated (0.82 and 0.83, respectively); statistically significant differences between females and males, and a high association between the metrics and sex, were identified. Posterior probabilities allow reliable estimations for all the measurements, excepting those between 31.0 and 31.5 mm, and the procedures that show the highest accuracy are those proposed by Seidemann et al. (1998), Curate et al. (2016), and Luna et al.(2021). Adult sex estimation from in a contemporary osteological sample from Buenos Aires, Argentina, with frequencies and probabilities between 0.82 and 0.83 for both sexes. The validation procedures implemented in this study highlight both the need to test quantitative models generated from diverse contemporary human populations, and the value of SID for obtaining reliable adult sex estimates, as they improve the quality of the biological profiles obtained in forensic contexts.

**Key points**

- Supero-inferior femoral neck diameter (SID) is relevant as a sex predictor due to its high preservation and dimorphic expression, especially in contexts of high fragmentation and commingling.
- The study validated the existing functions of SID by calculating the frequencies of correct estimations for males, females, and both sexes.
- The Bayesian approach yielded the best results, as posterior probabilities offered reliable estimations for almost all the measurements.
- The study highlighted that SID showed sexual dimorphism in this population.

## Introduction

In order for bioarchaeological investigations and forensic reports to be considered reliable, they must necessarily be based on the generation of adequate inferences regarding the sex and age-at-death of the individuals analyzed. The development and testing of appropriate procedures for sex estimation have become an extremely relevant aim in recent decades as contributions that help improve the conclusions obtained in the forensic disciplines, due to several factors. A great diversity of natural and cultural factors, both occurring during and after the process of deposition of the remains, can lead to the alteration, deterioration, and mixing of the recovered remains. This often severely limits the applicability of the most commonly used methods, which rely on morphological variables of the coxal bone and the skull, as well as the diameters of the femur and humeral heads, while hindering the development of a multifactorial approach that generally provides highly reliable results and is preferable to single-variable analysis for sex estimation [1, 2]. As a consequence, a large number of methods that evaluate the skeleton and the dentition from a quantitative perspective have been generated and tested in search of alternatives to obtain reliable sexual information ([3–15], among many others). These procedures must necessarily consider that both phenotypic variation in robusticity and size, and the secular trends, may influence sexual dimorphism, which can have significant consequences on the results obtained if not evaluated during the selection stage [16–21]. This is particularly important to develop the testing of the methods selected because the human remains analyzed to generate them differ from the reference sample in terms of population affinity, provenience, and/or chronology. The study of commingled, isolated, and/or deteriorated bone assemblages greatly benefits from the application of appropriate metric sex estimation methods; they are of great importance as they provide additional analysis strategies that can offer highly relevant information in cases where the techniques selected for application in simple and well-preserved burials cannot be used.

As recurrently stated by numerous previous studies [22–24], the femur has received considerable attention for conducting sexual estimations in adult individuals, because it offers several advantages over other skeletal elements. This element displays considerable sexual dimorphism due to its biomechanical connection with the pelvis, making it the most valuable bone for adult sex estimation, given female adaptation for childbirth [2, 22, 23, 25]. Moreover, the femur is generally better preserved compared to other skeletal elements due to its high proportion of cortical structure and being surrounded by a substantial muscle mass [22, 26]. Although many studies indicate relatively high levels of sexual dimorphism, primarily in the head of the femur, interpopulation variations of different magnitudes are usually identified [2, 27, 28]. The most analyzed variable is the head diameter, while others, such as the maximum femoral length, the circumference, the antero-posterior and mediolateral diameters at the midshaft, and the distal breadth, have received less attention [23, 25, 29–46].

The proximal end of the femur is particularly valuable due to its close biomechanical relationship with the pelvic bones and its important function in weight transmission within the body [1, 23, 47]. The minimum supero-inferior femoral neck diameter (SID) has received limited attention in previous studies. However, given that the proximal region of the femur is generally better preserved compared to the distal epiphysis and has shown significant sexual dimorphism [29, 38, 48, 49], it is very valuable for sex estimation in forensic anthropology and bioarchaeology [17, 22, 31, 35, 44, 50–52]. This is especially relevant for contributing to the accuracy of laboratory protocols when dealing with deteriorated, disturbed, commingled, and/or isolated human remains, especially as the femoral neck tends to have lower frequencies of postmortem damage compared to the femoral head.

Seidemann et al. [52] firstly proposed the utility of SID for sex estimation in two modern samples of African and European descendants included in the Hamann-Todd collection. The discriminant functions correctly estimated sex between 87% and 92%. Only the formula that included European descendants is relevant for this research; it is comprised of 50 males and 50 females, with a percentage of correct estimation of 92% (*F* = 94.0%; *M* = 90.0%). One year later, Stojanowski and Seidemann [53] analyzed a sample of 20th Century Native Americans, African Americans and European descendants from the Documented Collection at the University of New Mexico (49 females and 94 males). They evaluated the accuracy of the discriminant functions proposed by Seidemann et al. [52], with 83% to 97% correctly sexed, and created a new formula for European descendants (32 females and 62 males) that correctly classified 84% of the individuals (*F* = 91.0%; *M* = 80.0%). Posteriorly, Alunni-Perret et al. [54] recorded a modern sample of 70 French individuals (35 females and 35 males) born during the 20th Century and included in the Nice Collection, with 90.1% correctly sexed.

The research developed by Curate et al. [17] is especially important for the present research because it included two different samples from Portuguese reference skeletal collections. A sample of 252 individuals (114 females and 138 males) that come from the Luís Lopes Collection (National History Museum of Lisbon) and died between 1891 and 1959 was used to generate the functions, while another sample from the Coimbra Identified Skeletal Collection (University of Coimbra), comprised by 196 individuals (98 females and 98 males) who died between 1910 and 1936, was studied to validate the predictive models. The logistic regression created correctly estimated sex in 82.7% of the individuals from the test sample (*F* = 73.5%; *M* = 91.8%). The authors also developed a free online programme (osteomics.com/SeuPF/) that calculates the probabilities that the regression results point to a male or a female. More recently, Carvallo and Retamal [47] proposed a series of new uni- and multivariate logistic regressions concerning the proximal end of the femur in a modern Chilean sample of 270 individuals (135 females and 135 males; 200 for the development of the functions and 70 for validation) who died between 1950 and 1973, from the Subactual Osteological Collection of Santiago de Chile (Colección Osteológica Subactual de Santiago; COSS), housed in the Department of Anthropology, University of Chile. The model based only on SID correctly classified 85.7% of the individuals (*F* = 85.7%; *M* = 85.7%). Finally, Luna et al. [55] generated a discriminant function and a binary logistic regression equation studying a sample of 164 individuals (70 females and 94 males) from the Chacarita reference osteological collection (contemporary Chacarita Cemetery, Buenos Aires City, Argentina), who died between 1992 and 2015. Around 89.90% of the females and 90.23% of the males were correctly classified using the discriminant functions, while the values from the logistic regression were 90.56% and 91.06%, respectively. In addition, the authors calculated a sectioning point from the direct measurements (SP = 30.86 mm), that best discriminate between sexes, and developed a Naïve Bayesian approach for posterior probabilities calculation. Results obtained showed very good values of sex estimation when the direct SP is considered (*F* = 85.0%; *M* = 90.0%), and also after the application of the probabilistic procedure (*F* = 0.93; *M* = 0.91).

Forensic anthropological investigations have experienced continuous and accelerated growth, particularly since the late 20th Century. Nowadays, the discipline has achieved highly relevant results in complex recovery situations that were challenging to analyze in the recent past. The large number of available metric methods for sexual estimation in samples from different provenience worldwide highlights the need for a detailed assessment of their reliability when applied to samples of different origins than those used to develop the formulae. Obtaining satisfactory results in this regard may render the development of new local formulae unnecessary. Consequently, this research aims to identify the discriminatory potential of SID for sex estimation and to test different formulae and mathematical procedures currently available in the forensic literature for sexual estimation recording the SID as a single variable, on a sample of contemporary human remains from the 21st Century Identified Skeletal Collection (University of Coimbra, Portugal). This will help identify which methods offer adequate results for application in modern Portuguese forensic contexts, thereby contributing to the generation of more reliable biological profiles. The relevance of addressing the contribution of univariate quantitative strategies is emphasized, not only given their potential applicability to fragmented and deteriorated bones, but also considering their ease and speed in obtaining the estimates.

## Sample and methods

The 21st Century Identified Skeletal Collection is housed in the Laboratory of Forensic Anthropology, Department of Life Sciences at the University of Coimbra, Portugal, and includes 302 not claimed male and female adult skeletons, mostly elderly (only 12.25% are younger than 61 years old). These individuals died between 1982 and 2012, and were exhumed between 1999 and 2016 from the Capuchos cemetery of Santarém city [56, 57]. Two hundred and ninety-five individuals (157 females (53.22%) and 138 males (46.78%)) between 25 and 101 years old (mean age: female, 81.07 years; male, 73.19 years; overall = 77.27 years) were available for analysis, comprising a total of 520 femora of both sides (252 rights, 268 lefts; 238 of males and 282 of females) (Tables 1 and 2). Table 2 shows that the ages-at-death for more than half of the individuals in the sample range from 70 to 89 years (*n* = 174; 58.98%), with a low representation among those younger than 49 years old (*n* = 19; 6.44%). The present research received ethical and procedural approval from the Scientific Commission of the 21st Century Identified Skeletal Collection.

**Table 1:** Distribution of supero-inferior femoral neck diameter (SID) analyzed based on sex and side.

Sex	Number of individuals (*N*)	Right (*n*, %)	Left (*n*, %)	Total (*N*, %)
Female	157	137, 26.35	145, 27.88	282, 54.23
Male	138	115, 22.11	123, 23.65	238, 45.77
Total	295	252, 48.46	268, 51.53	520, 100

Numbers are rounded so the percentages may not add up to 100%.

**Table 2:** Distribution of supero-inferior femoral neck diameter (SID) analyzed based on sex and age-at-death.

Sex	25–29 years (*n*, %)	30–39 years (*n*, %)	40–49 years (*n*, %)	50–59 years (*n*, %)	60–69 years (*n*, %)	70–79 years (*n*, %)	80–89 years (*n*, %)	90–101 years (*n*, %)	Total (*n*, %)
Female	1, 0.34	2, 0.68	5, 1.70	4, 1.36	14, 4.75	31, 10.51	61, 20.68	39, 13.22	157, 53.22
Male	3, 1.02	5, 1.70	3, 1.02	6, 2.03	26, 8.81	40, 13.56	42, 14.24	13, 4.41	138, 46.78
Total	4, 1.36	7, 2.37	8, 2.71	10, 3.39	40, 13.56	71, 24.07	103, 34.92	52, 17.63	295, 100

Numbers are rounded so the percentages may not add up to 100%.

Only femora without any pathological or taphonomic alterations in the neck region were included in the sample. The SID was recorded twice, without previous knowledge of the sex and age of the individuals, on the femoral neck area, considering the minimum diameter in a supero-inferior direction [52]. Each measurement was carried out at least 2 weeks apart and obtained to the nearest hundredth of a millimeter using a sliding digital caliper. The data recorded were used to calculate the intraobserver error through the relative Technical Error of Measurement (rTEM) [58, 59].

Descriptive statistics (minimum and maximum values, mean, standard deviation, and percentiles) were calculated separately for each sex and side. Since male and female sample sizes are different, using the average between both centroid groups for obtaining SP can lead to significant bias in favour of the smaller group. Consequently, it was obtained by applying the following formula, which considers the differences in group sizes:


$$ \mathrm{SP}=\left(\left({n}_{\mathrm{f}}\times{\mathrm{a}}_{\mathrm{f}}\right)+\left({n}_{\mathrm{m}}\times{\mathrm{a}}_{\mathrm{m}}\right)\right)/\left({n}_{\mathrm{f}+}{n}_{\mathrm{m}}\right) $$

where *n*_f_ and *n*_m_ represent the sample sizes of each sex, and *a*_f_ and *a*_m_ represent the averages of the values for each group. In a second step, the significance of the differences and the strength of the association between the measurements and sex were assessed using the Kolmogorov–Smirnov test (*Z*) (*α* = 0.05) and Eta (*η*), respectively; the variation by side was also calculated using *Z*. The incidence of age-at-death on the estimations was calculated for each sex, with the sample divided into four age groups (≤40, 41–60, 61–80, and ≥ 81 years old) by means of both Pearson correlation coefficient (*r*) for comparison between the documented age and SID, and *Z* for the evaluation of the statistical significance among cohorts. Finally, sexual dimorphism was evaluated through the estimation of the relative distance between the means of each sex, as follows: SD = ($\overline{x}$*_M_*−$\overline{x}$*_F_*)/$ \, \overline{x}$*_F_* × 100% [60], where $\overline{x}$ is the average of SID for each sex.

A total of seven formulae and one calculation based on direct measurements were used in this research, previously generated by Seidemann et al. [52] (formula for European descendants), Stojanowski and Seidemann [53], Alunni-Perret et al. [54], Curate et al. [17], Carvallo and Retamal [47], and Luna et al. [55]. These correspond to all available in the literature for samples composed by individuals of European ancestors, which analyze SID isolately (Table 3). After applying the functions, the sex for each individual was estimated considering the sectioning point provided by each paper, and the frequencies of correct estimation (*P*(A|B)) for males, females and both sexes were calculated, showing the overall success rate for the sample. The likelihoods of correct allocation (*P*(B|A)) were also estimated [61] as the proportion of females relative to the number of individuals located below the sectioning point, and the same for males above that threshold [62].

**Table 3:** Descriptive statistics, original formulae used in the present research, and percentages of original correct estimations.

References	Collection	Male				Male				Formulae	SP	%		
		*n*	Mean±SD	Min	Max	*n*	Mean±SD	Min	Max			Female	Male	Overall
[52]	Hamann-Todd	50	27.8±1.7	24.5	31.8	50	33.5±2.2	29.4	39.6	X=0.496×SID-15.163	0	94.0	90.0	92.0
[53]	University of New Mexico	32	28.0±1.9	NA	NA	62	33.9±2.9	NA	NA	X= 0.387×SID-12.462	0	91.0	80.0	84.0
[54]	Nice	35	30.8±2.2	26.3	30.7	35	35.1±2.4	29.5	35.4	X=0.505×SID-17.136	0	NA	NA	90.1
[17]^*^	Luís Lopes Collection	114	29.4±2.1	NA	NA	138	34.3±2.7	NA	NA	X=0.968×SID-30.445	0	NA	NA	82.7
[47]	COSS	100	27.31±1.9	NA	NA	100	31.78±2.2	NA	NA	X=1.115×SID-32.727	0	85.7	85.7	85.7
[55]	Chacarita Collection	58	29.4±2.9	21.7	34.6	87	34.3±2.6	27.3	40.7	X=0.478×SID-12.798	−0.2	89.9	90.2	90.1
										X=1/[1+*e*^-(21.921-0.701×SID)^]	0.5	90.5	91.0	90.7
										Direct measurement	30.86^#^	85.3	90.6	87.7

SD: standard deviation; SP: sectioning point; COSS: Colección Osteológica Subactual de Santiago de Chile (Subactual Osteological Collection of Santiago de Chile); NA: not available; SID: supero-inferior femoral neck diameter; ^*^: an online app that estimates sex and posterior probabilities from this formula is available at osteomics.com/SeuPF/;  ^#^: value in mm.

Finally, the posterior probabilities were estimated using Bayesian statistics, dividing the total range of measurements in 1-mm sections. Bayesian probabilities are used as a method to quantify uncertainty about the likelihood of an event or hypothesis based on both prior knowledge and new evidence. This approach involves updating the previously known data as more information is obtained, combining the initial assumptions (prior probabilities) with observed data (likelihood) to calculate a more accurate (posterior) probability. In the present research, the procedure evaluates the chances that an individual with a value higher than each the sectioning point considered, be a male, or, on the contrary, that one with a lower value, be a female, using the traditional Bayesian theorem:


$$ P\left(\mathrm{B}|\mathrm{A}\right)=\frac{P\left(\mathrm{B}\right)\;P\left(\mathrm{A}|\mathrm{B}\right)}{\sum P\left(\mathrm{A}|\mathrm{B}\right)\;P(B)} $$

where *P*(A|B) is the likelihood (the probability of event A being true, given event B has occurred), *P*(B) is the prior (probability of event B being true), ∑ *P*(A|B) *P*(B), or *P*(A), is the probability of event A being true, and *P*(B|A) is the posterior probability, or the probability of B being true, given that A is true. The outcome of the equation is the probability of sex for a given measure. The prior used in this case corresponds to the frequencies of the bones recorded, by sex [61–64]. SPSS 16.0 (IBM Corporation, Armonk, NY, USA) and PAST 3.15 (University of Oslo, Oslo, Norway) programmes were used for the statistical procedures.

## Results

**Figure 1:**
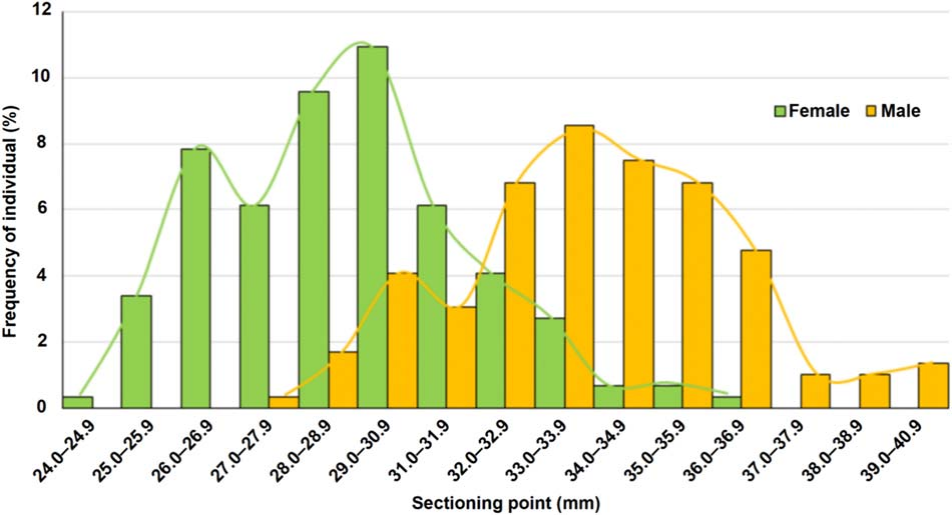
Frequencies of individuals according to SID dimensions, by sex.

Figure 1 provides the frequency distribution of individuals according to SID dimensions, with divergent values by sex and only a few measurements shared by both in the middle of the graphic. The value of rTEM obtained was 0.93%, which means very low magnitudes of intraobserver error and high replicability of the measure. Descriptive statistics are shown in Table 4, for sex of the individuals and side of the femora. After minimum and maximum values, very low overlapping is inferred, with clearly different means. Female measurements are consistently lower than those for males, indicating SID is a dimorphic variable. Taking into account the direct sectioning point (SP = 30.58 mm), the frequencies of cases correctly estimated are 0.80 for females and 0.84 for males (Overall = 0.82), while the probabilities of sexual allocation are 0.84 for females and 0.82 for males (Overall = 0.83). Statistically significant differences between sexes (*Z* = 5.68; *P* = 0.00) and a high association between the metrics and sex (*η =* 0.89) were identified.

**Table 4:** Descriptive statistics of supero-inferior femoral neck diameter (SID) analyzed, frequencies of cases correctly estimated (*P*(A|B)) and probabilities of sexual allocation (*P*(B|A)).

		*N*	Min	Max	Mean ± SD	25th percentile	50th percentile	75th percentile	*P*(A|B)	*P*(B|A)
Female	Left	145	24.58	35.38	28.87 ± 4.46	27.25	28.76	30.11	0.80	0.84
	Right	137	25.01	35.02	28.74 ± 4.95	27.39	28.74	30.08		
	Both	282	24.58	35.38	28.79 ± 4.72	27.33	28.75	30.10		
Male	Left	123	27.29	38.95	33.02 ± 5.19	31.65	32.97	34.39	0.84	0.82
	Right	115	27.03	38.79	32.72 ± 5.90	31.14	32.66	34.51		
	Both	238	27.03	38.95	32.87 ± 5.54	31.42	32.75	34.43		
Overall	SP = 30.58 mm								0.82	0.83

SD: standard deviation; SP: sectioning point.

**Figure 2:**
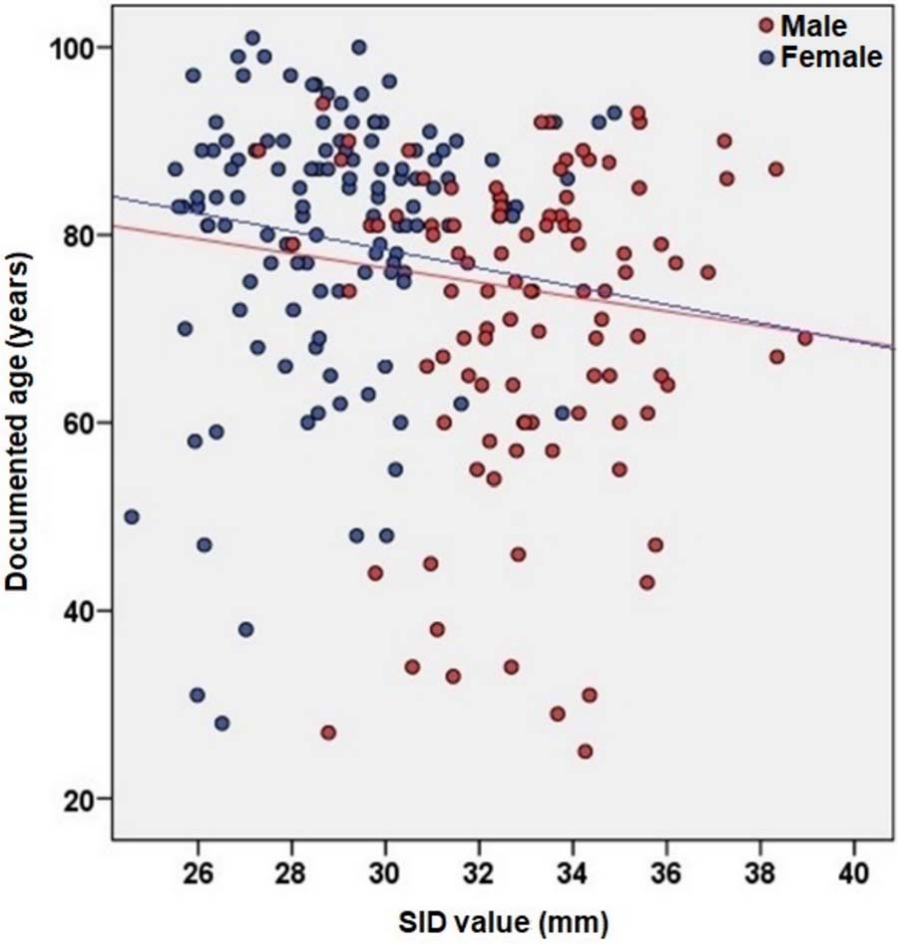
Variation of supero-inferior femoral neck diameter (SID) with age-at-death, by sex.

Right and left SID differences are not statistically significant for both sexes (*F*: *Z* = 0.51, *P* = 0.96; *M*: *Z* = 0.42, *P* = 0.99; total: *Z* = 0.47, *P* = 0.98), so it can be stated that side does not influence the dimorphic patterns observed. Moreover, the incidence of age-at-death on the estimations is negligible in all cohorts, taking into account the results about correlations (*r*; Table 5) and statistical significances (*Z*; Table 6). The trends of SID measurements with age are shown in Figure 2. Moreover, the percentage of sexual dimorphism [60] is 13.68%.

**Table 5:** Pearson coefficient of correlation (*r*) comparisons between supero-inferior femoral neck diameter (SID) and the documented age-at-death.

	≤40 years	41–60 years	61–80 years	≥81 years	Total
Female	0.25 (0.29)	−0.28 (0.31)	0.54 (0.10)	0.45 (0.14)	0.38 (0.15)
Male	0.32 (0.36)	−0.13 (0.39)	0.36 (0.15)	0.16 (0.22)	0.11 (0.36)
Total	0.28 (0.31)	−0.21 (0.34)	0.42 (0.12)	0.34 (0.17)	0.22 (0.23)

**Table 6:** Statistical significance among cohorts, by sex and age-at-death (*Z* and *P*).

		≤40 years		41–60 years		61–80 years		≥81 years		Total	
		Male	Female	Male	Female	Male	Female	Male	Female	Male	Female
≤40 years	Male	-	0.57	0.41	0.22	0.37	0.62	0.60	0.46	0.61	0.59
	Female	0.82	-	0.38	0.46	0.26	0.55	0.60	0.47	0.32	0.37
41–60 years	Male	0.88	0.91	-	0.27	0.37	0.62	0.54	0.26	0.50	0.44
	Female	0.96	0.86	0.93	-	0.37	0.40	0.13	0.54	0.52	0.46
61–80 years	Male	0.80	0.87	0.81	0.82	-	0.22	0.31	0.52	0.33	0.41
	Female	0.65	0.60	0.64	0.89	0.95	-	0.42	0.60	0.37	0.19
≥81 years	Male	0.67	0.66	0.70	0.99	0.81	0.90	-	0.20	0.22	0.27
	Female	0.88	0.88	0.97	0.72	0.70	0.67	0.98	-	0.27	0.30
Total	Male	0.75	0.86	0.84	0.83	0.87	0.89	0.95	0.94	-	0.49
	Female	0.79	0.91	0.88	0.88	0.89	0.96	0.94	0.91	0.88	-

Values of *Z* are grouped in the upper right area of the table, and the probabilities, on the lower left.

**Figure 3:**
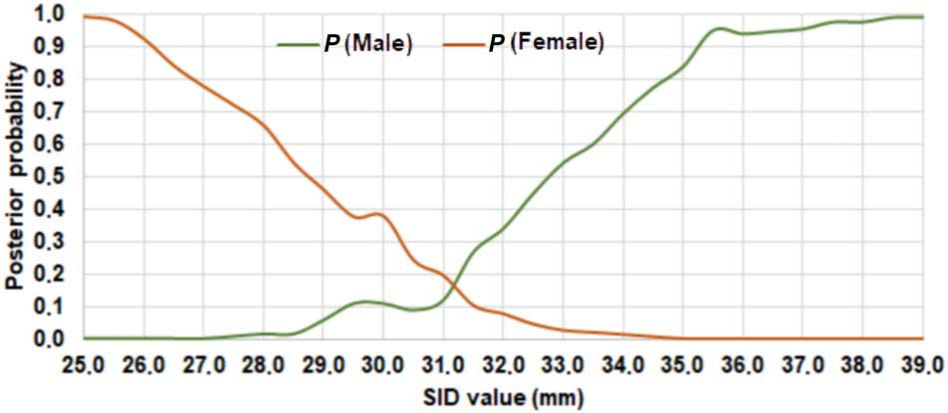
Female and male posterior probabilities for different supero-inferior femoral neck diameter (SID) values.

The results obtained after the application of the procedures proposed by Seidemann et al. [52], Stojanowski and Seidemann [53], Alunni-Perret et al. [54], Curate et al. [17], Carvallo and Retamal [47], and Luna et al. [55] are given in Table 7. The percentages of correct estimations for both sexes range between 67.12% (formulae by [47, 54]) and 83.05% (formula by [17] and direct measurement by [55]). It is highlighted that the data obtained from the formula generated by Seidemann et al. [52] also show high percentages of correct estimations (82.03%). Those for females vary between 60.51% (discriminant function by [55]) and 98.09% (formula by [54]), and those for males, between 31.88% (formula by [54]) and 84.06% (formula by [52]). On the other hand, the probabilities of correct allocation for both sexes range between 0.67 (formulae by [47, 54]) and 0.83 (formula by [17] and direct measurement by [55]). If only females are considered, the values vary from 0.62 (formula by [54]) to 0.86 (formula by [52]), and concerning males, between 0.59 (formula by [47]) and 0.94 [54]. Finally, the posterior probabilities obtained after the application of the Bayesian theorem (Table 8; Figure 3) indicate that the comparison of male and female values allows adequate sex estimations for all the measurements, excepting those between 31.0 and 31.5 mm. In these cases, the results are much more doubtful.

**Table 7:** Percentages of correct estimations (*P*(A|B)) and probabilities (*P*(B|A)) for males, females, and the whole sample, considering the formulae previously generated by Seidemann et al. [52], Stojanowski and Seidemann [53], Alunni-Perret et al. [54], Curate et al. [17], Carvallo and Retamal [47], and Luna et al. [55].

Reference		*P*(A|B)						*P*(B|A)					
		Female		Male		Overall		Female		Male		Overall	
		*n*/*N*	%	*n*/*N*	%	*n*/*N*	%	*n*/*N*	*P*	*n*/*N*	*P*	*n*/*N*	*P*
[52]		126/157	80.25	116/138	84.06	242/295	82.03	126/147	0.86	116/148	0.78	242/295	0.82
[53]		144/157	91.72	86/138	62.32	230/295	77.97	144/194	0.74	86/99	0.87	230/295	0.78
[54]		154/157	98.09	44/138	31.88	198/295	67.12	154/248	0.62	44/47	0.94	198/295	0.67
[17]^*^		139/157	88.54	106/138	76.81	245/295	83.05	139/170	0.82	106/125	0.85	245/295	0.83
[47]		100/157	63.69	98/138	71.01	198/295	67.12	100/128	0.78	98/167	0.59	198/295	0.67
[55]	DF	95/157	60.51	107/138	77.54	202/295	68.47	95/131	0.73	107/164	0.65	202/295	0.68
	LR	125/157	79.62	109/138	78.99	234/295	79.32	125/156	0.80	109/139	0.78	234/295	0.79
	DM	131/157	83.44	114/138	82.61	245/295	83.05	131/155	0.85	114/140	0.81	245/295	0.83

DF: discriminant function; LR: logistic regression; DM: direct measurement; ^*^: values estimated from osteomics.com/SeuPF/.

**Table 8:** Female and male posterior probabilities for different supero-inferior femoral neck diameter (SID) measurements.

SP (mm)	*P* (Male)	*P* (Female)	SP (mm)	*P* (Male)	*P* (Female)	SP (mm)	*P* (Male)	*P* (Female)
25.0	0.00	0.99	30.0	0.11	0.38	35.0	0.84	0.00
25.5	0.00	0.98	30.5	0.09	0.24	35.5	0.95	0.00
26.0	0.00	0.92	31.0	0.12	0.19	36.0	0.94	0.00
26.5	0.00	0.84	31.5	0.27	0.10	36.5	0.95	0.00
27.0	0.00	0.78	32.0	0.34	0.08	37.0	0.96	0.00
27.5	0.01	0.72	32.5	0.45	0.04	37.5	0.98	0.00
28.0	0.01	0.66	33.0	0.54	0.03	38.0	0.98	0.00
28.5	0.01	0.54	33.5	0.60	0.02	38.5	0.99	0.00
29.0	0.06	0.46	34.0	0.70	0.01	39.0	0.99	0.00
29.5	0.11	0.38	34.5	0.78	0.01			

SP: sectioning point.

## Discussion and conclusions

The analysis of mixed, fragmented, and deteriorated human skeletal remains has undergone significant development in recent years in the field of bioarchaeology and forensic anthropology, resulting in a notable improvement in the quality of results related to the biological profile and identification. The development and validation of specific procedures for reliable sex estimation, especially in situations where a multifactorial approach is not possible, are of vital importance to contribute to the resolution of such cases [1, 2, 65]. The femoral neck is particularly relevant as one of the best-preserved anatomical portions of the human skeleton, even in cases of intentional cremation or intense impact of natural taphonomic agents. In this regard, the testing of the available methodological proposals allows establishing which ones can be confidently applied in forensic scenarios, in this case, involving contemporary Portuguese individuals.

Previous studies that addressed sex estimation using SID provided satisfactory results using samples from the same origin as those recorded for generating the method [17, 47, 52–55], which demonstrate the usefulness of the technique. However, when these metric procedures are applied in individuals from different geographical or temporal origins, the results tend to be less reliable; for that reason, it is extremely important that they are tested in different contexts to evaluate their relevance for forensic applications. The present research indicates that intraobserver error included during the recording process is negligible and that the real values were not significantly affected. It also underlines that SID is not highly correlated with age-at-death (Figure 2; Table 5) and do not significantly differ by side. Overlapping between sexes is low (Figure 1; Table 4) and statistically significant differences between females and males were identified, together with a high association between metrics and sex, and a high value of sexual dimorphism. All this information points that SID is a skeletal area highly relevant for sex estimation in 21st Century Portuguese populations.

When the value of sexual dimorphism obtained (SD = 13.68%) is compared with those of the samples from which the tested methods were created (Table 9), it is possible to identify that it is the lowest; only that from Alunni-Perret et al. [54] is similar (13.74%), while the other ones range between 16.02% [55] and 20.35% [52, 53]. Moreover, the sample analyzed by Curate et al. [17], from the same origin but including individuals dead during the end of the 19th Century and the beginning of the 20th Century, shows a value of 16.55%, which highlights the influence of secular changes in bone dimensions, and especially in sexual dimorphism patterns, on generating an unbalanced classification between sexes [18, 66]. Despite the fact that the degree of sexual dimorphism appears to diminish when comparing both samples, the application of the procedures proposed by Curate et al. [17] offers the highest percentages of correctly estimated cases and probabilities of correct allocation, both for each sex and considering the sample as a whole (*F*: *P* = 0.82, 88.54%; *M*: *P* = 0.85, 76.81%; overall: *P* = 0.83, 83.05%; Table 7). This indicates that the magnitude of the differences in sexual dimorphism did not significantly influence the results and, consequently, that the method can be confidently used in contemporary samples from the same origin.

**Table 9:** Values of sexual dimorphism obtained according to the formula by Garn et al. [60] for the samples from which the formulae were generated.

Reference	$\overline{x}$ Female (mm)	$\overline{x}$ Male (mm)	SD (%)
[52]; [53]	27.86	33.53	20.35
[54]	30.85	35.09	13.74
[17]	29.43	34.31	16.55
[47]	27.31	31.79	16.39
[55]	29.63	34.30	16.02
Present study	28.94	32.90	13.68

SD: sexual dimorphism. The values were calculated based on the published averages for females and males.

On the other hand, high percentages and probabilities were obtained when the sectioning point obtained by Luna et al. [55] in the analysis of the Chacarita Collection (SP = 30.86), which includes skeletons of contemporary individuals from the city of Buenos Aires (Argentina), is considered (*F*: *P* = 0.85, 83.44%; *M*: *P* = 0.81, 82.61%; overall: *P* = 0.83, 83.05%; Table 7). These results are striking considering that direct approximations of measurements generally show a less effective performance for sex estimation. In any case, the results obtained from the logistic regression from the same paper are also satisfactory (*F*: *P* = 0.80, 79.62%; *M*: *P* = 0.78, 78.99%; overall: *P* = 0.79, 79.32%; Table 7). These trends can be attributed to the processes of admixture that occurred during the last 400 years in Argentina, primarily those resulting from the massive immigration that took place in Buenos Aires and its surroundings during the second half of the 19th Century and early 20th Century from various Western European countries (mainly Italy and Spain, but also the UK, Ireland, and Portugal). As the dimorphism identified is closely related to the phenotypic characteristics of those populations [55], it is possible to propose that the procedures generated from the analysis of the Argentinian sample would be suitable for sex estimation of modern Portuguese individuals.

The formula proposed by Seidemann et al. [52], based on the analysis of individuals of European ancestry from the Hamann Todd collection, also yielded satisfactory results (*F*: *P* = 0.86, 80.25%; *M*: *P* = 0.78, 84.06%; overall: *P* = 0.82, 82.03%; Table 7). In contrast, the estimates derived from the other three methods [47, 53, 54] show different trends. The discriminant function by Stojanowski and Seidemann [53], generated from the study of individuals with European, African, Native American, and unknown ancestry from the University of New Mexico collection, offered acceptable estimations when analyzing both sexes together (77.97%; *P* = 0.78). However, it only provided high values of correct estimations for females (*F* = 91.72%; *M* = 62.32%) and probabilities for male individuals (*F*: *P* = 0.74; *M*: *P* = 0.87; Table 7). This means a significant weakness of this formula for its application in the Portuguese sample, resulting in a considerable number of male individuals being misclassified as females.

A similar pattern is observed for the results obtained using the formula by Alunni-Perret et al. [54], developed with individuals of European ancestry from the Nice Collection (France). In this case, the discrepancies between the values obtained for each sex are even greater, with overall unsatisfactory results (*F*: *P* = 0.62, 98.09%; *M*: *P* = 0.94, 31.88%; overall: *P* = 0.67, 67.12%; Table 7). Finally, the formula proposed by Carvallo and Retamal [47], based on the study of Chilean individuals who died during the second half of the 21st Century, is also inadequate for use in the analyzed sample, as the classifications generally resulted in unsatisfactory outcomes (*F*: *P* = 0.78, 63.69%; *M*: *P* = 0.59, 71.01%; total: *P* = 0.67, 67.12%; Table 7). In this case, a high component of admixture between European migrants and indigenous populations is likely to be influencing the results.

After the comparison of all the results, it is possible to affirm that the procedure that shows the highest accuracy is that of Curate et al. [17], followed by Luna et al. [55] direct SP, Seidemann et al. [52] discriminant function and Luna et al. [55] logistic regression. It was not possible to identify a better performance for either sex, as Seidemann et al. [52] formula offers much better results for males, that of Curate et al. [17], higher percentages for females, and both the direct SP and the logistic regression proposed by Luna et al. [55], similar percentages for both sexes. In contrast, the remaining functions should not be used since they do not provide sufficiently high percentages of correct estimations, at least in one of the sexes. These trends emphasize the importance of considering the population origin of the individuals to be analyzed when selecting the most appropriate procedures to apply in local cases, given the worldwide phenotypic variation in quantitative variables observed among current human populations related to sexual dimorphism [41].

Furthermore, the comparison of the posterior probability values from Table 8 allows for a reliable sex estimation in this sample. The procedure offers a robust alternative to the frequentist perspective because of its statistical rigor, as it quickly and reliably allocated most of the individuals, considering all possible measurements within the observed range of variation. In this case, SID values lower than 31.0 mm indicate that the individual has a high probability of being female, while measurements greater than 31.5 suggest that the individual would be male (Figure 3).

In conclusion, as stated in several previous papers [17, 18, 22, 25, 32, 35, 38, 48, 49, 67], the proximal region of the femur is much valuable for sex estimation in both bioarchaeological and forensic research, especially when the pelvic bones are not well-preserved. This research highlights the value of sexual dimorphism of SID in obtaining reliable sex information in contemporary Portuguese human remains, especially in contexts of high fragmentation and commingling, if possible, in conjunction with other skeletal indicators. This is particularly relevant since the femoral neck is usually well preserved, in contrast to the maximum diameters of the femoral head. Moreover, quantitative data are less subjective compared to visually assessment of morphological variables, leading to more accurate results [68]. Finally, as sexual dimorphism commonly differs among skeletal populations, mainly conditioned by genetic, hormonal and biomechanical aspects, as well as by nutritional and/or or socioeconomic status [18], it is very important both to generate population-specific methods for sex estimation and to test them in other spatial and/or temporal skeletal samples [1]. The assessment of the procedures analyzed in the present research on contemporary samples of both close (e.g. Western Europe) and distant origin (but from populations that underwent intense migratory processes from European populations, such as those of North and South America), constitutes a necessary step to identify the relevance of their application in forensic cases from different regions of the world.

## References

[1] Curate F . The estimation of sex of human skeletal remains in the Portuguese identified collections: history and prospects. Forensic Sci. 2022;2:272–286.

[2] Klales AR . Sex estimation of the human skeleton. New York (NY): Academic Press; 2020.

[3] Albanese J, Cardoso H, Saunders S. Universal methodology for developing univariate simple-specific sex determination methods: an example using the epicondylar breadth of the humerus. J Archaeol Sci. 2005;32:143–152.

[4] Celbis O, Agritmis H. Estimation of stature and determination of sex from radial and ulnar bone lengths in a Turkish corpse sample. Forensic Sci Int. 2006;158:135–139. 15990262 10.1016/j.forsciint.2005.05.016

[5] Karakostis F, Zorba E, Moraitis K. Osteometric sex determination using proximal foot phalanges from a documented human skeletal collection. Anthropolischer Anzeiger. 2014;71:403–427. 10.1127/0003-5548/2014/0423 25774700

[6] Luna L . Canine sex estimation and sexual dimorphism in the collection of identified skeletons of the University of Coimbra, with an application in a Roman cemetery from Faro. Portugal Inter J Osteoarcheol. 2019;29:260–272.

[7] Lynch J, Cross P, Heaton V. Sexual dimorphism of the first rib: a comparative approach using metric and geometric morphometric analyses. J Forensic Sci. 2017;62:1251–1258. 28168691 10.1111/1556-4029.13421

[8] Maranho R, Ferreira MT, Curate F. Sexual dimorphism of the human scapula: a geometric morphometrics study in two Portuguese reference skeletal samples. Forensic Sci. 2022;2:780–794.

[9] Mokoena P, Billings B, Gibbon V, et al. Development of discriminant functions to estimate sex in upper limb bones for mixed ancestry South Africans. Science and Justice. 2019;59:660–666. 31606104 10.1016/j.scijus.2019.06.007

[10] Partido NM, Monge Calleja Á, Ferreira MT, et al. Validation of discriminant functions from the rib necks in two Portuguese adults identified populations. Int J Leg Med. 2023;137:851–861. 10.1007/s00414-023-02957-8 36719511

[11] Roggio C, Magalhães BM, Santos AL. Adult sex estimation based on the 12th thoracic and 1st lumbar vertebrae from a Portuguese contemporary population: effects of degenerative lesions and comparison of accuracy with other skeletal areas. La Revue de Médecine Légale. 2023;14:100402.

[12] Scheuer J, Elkington N. Sex determination from metacarpals and the first proximal phalanx. J Forensic Sci. 1993;38:769–778. 8354997

[13] Stock M . Analyses of the postcranial skeleton for sex estimation. In: Klales AR, editor. Sex estimation of the human skeleton. New York (NY): Academic Press; 2020. p. 113–130.

[14] Uysal Ramadan S, Türkmen N, Dolgun N, et al. Sex determination from measurements of the sternum and fourth rib using multislice computed tomography of the chest. Forensic Sci Int. 2010;197:120.e1–120.e5. 10.1016/j.forsciint.2009.12.049 20083365

[15] Viciano J, D'Anastasio R, Capasso L. Odontometric sex estimation on three populations of the Iron Age from Abruzzo region (Central-Southern Italy). Arch Oral Biol. 2015;60:100–115. 25285904 10.1016/j.archoralbio.2014.09.003

[16] Christensen AM, Passalacqua NV, Bartelink EJ. Forensic anthropology: current methods and practice. San Diego (CA): Elsevier, 2014.

[17] Curate F, Coelho J, Gonçalves D, et al. A method for sex estimation using the proximal femur. Forensic Sci Int. 2016;266:579.e1–579.e7. 10.1016/j.forsciint.2016.06.011 27373600

[18] Guyomarc’h P, Velemínská J, Sedlak P, et al. Impact of secular trends on sex assessment evaluated through femoral dimensions of the Czech population. Forensic Sci Int. 2016;262:284.e1–284.e6. 10.1016/j.forsciint.2016.02.042 26980521

[19] Langley N, Jantz R. Secular change. In: Klales AR, editor. Sex estimation of the human skeleton. New York (NY): Academic Press; 2020. p. 295–306.

[20] Steyn M, Patriquin M. Osteometric sex determination from the pelvis. Does population specificity matter? Forensic Sci Int. 2009;191:113.e1–113.e5. 10.1016/j.forsciint.2009.07.009 19665855

[21] Ubelaker D, DeGaglia C. Population variation in skeletal sexual dimorphism. Forensic Sci Int. 2017;278:407.e1–407.e7. 10.1016/j.forsciint.2017.06.012 28698063

[22] Albanese J, Eklics G, Tuck A. A metric method for sex determination using the hipbone and the femur. J Forensic Sci. 2003;53:1283–1288. 10.1111/j.1556-4029.2008.00855.x 18717754

[23] Asala S, Bidmos M, Dayal M. Discriminant function sexing of fragmentary femur of South African blacks. Forensic Sci Int. 2004;145:25–29. 15374591 10.1016/j.forsciint.2004.03.010

[24] Kim D, Kwak D, Han S. Sex determination using discriminant analysis of the medial and lateral condyles of the femur in Koreans. Forensic Sci Int. 2013;233:121–125. 24314510 10.1016/j.forsciint.2013.08.028

[25] Kranioti E, Vorniotakis N, Galiatsou C, et al. Sex identification and software development using digital femoral head radiographs. Forensic Sci Int. 2009;189:113.e1–113.e7. 10.1016/j.forsciint.2009.04.014 19443150

[26] Curate F, Umbelino C, Perinha A, et al. Sex determination from the femur in Portuguese populations with classical and machine-learning classifiers. J Forensic Leg Med. 2017;52:75–81. 28866285 10.1016/j.jflm.2017.08.011

[27] Frayer D, Wolpoff M. Sexual dimorphism. Ann Rev Anthropol. 1985;14:429–473.

[28] Hamilton ME . Sexual dimorphism in skeletal samples. In: Hall RL, editor. Sexual dimorphism in Homo sapiens: a question of size. New York (NY): Praeger; 1982. p. 107–163.

[29] Asala S, Mbajiorgu F, Papandro B. A comparative study of femoral head diameters and sex differentiation in Nigerians. Acta Anat. 1998;162:232–237. 9831772 10.1159/000046438

[30] Black T . A new method for assessing the sex of fragmentary skeletal remains: femoral shaft circumference. Am J Phys Anthropol. 1978;48:227–231. 637123 10.1002/ajpa.1330480217

[31] Brown R, Ubelaker D, Schanfield M. Evaluation of Purkait’s triangle method for determining sexual dimorphism. J Forensic Sci. 2007;52:553–556. 17456081 10.1111/j.1556-4029.2007.00423.x

[32] Colman K, Janssen M, Stull K, et al. Dutch population specific sex estimation formulae using the proximal femur. Forensic Sci Int. 2018;286:268.e1–268.e8. 10.1016/j.forsciint.2017.12.029 29548547

[33] du Jardin P, Ponsaillé J, Alunni-Perret V, et al. A comparison between neural network and other metric methods to determine sex from the upper femur in a modern French population. Forensic Sci Int. 2009;192:127.e1–127.e6. 10.1016/j.forsciint.2009.07.014 19733989

[34] Fliss B, Luethi M, Fuernstahl P, et al. CT-based sex estimation on human femora using statistical shape modeling. Am J Phys Anthropol. 2019;169:279–286. 30927271 10.1002/ajpa.23828

[35] Meeusen R, Christensen A, Hefner J. The use of femoral neck axis length to estimate sex and ancestry. J Forensic Sci. 2015;60:1300–1304. 26258403 10.1111/1556-4029.12820

[36] Milner G, Boldsen J. Humeral and femoral head diameters in recent White American skeletons. J Forensic Sci. 2012;57:35–40. 22074229 10.1111/j.1556-4029.2011.01953.x

[37] Ozer I, Katayama K. Sex determination using the femur in an ancient Japanese population. Coll Antropol. 2008;32:67–72. 18494190

[38] Purkait R . Triangle identified at the proximal end of femur: a new sex determinant. Forensic Sci Int. 2005;147:135–139. 15567617 10.1016/j.forsciint.2004.08.005

[39] Purkait R, Chandra H. A study of sexual variation in Indian femur. Forensic Sci Int. 2004;146:25–33. 15485718 10.1016/j.forsciint.2004.04.002

[40] Ranaweera L, Cabral E, Dissanayake V, et al. Estimation of sex from the osteometric measurements of the femur in a contemporary Sri Lankan population. Inter J Morphology. 2022;40:1009–1017.

[41] Ríos FL . Brief communication: sex determination accuracy of the minimum supero-inferior femoral neck diameter in a contemporary rural Guatemalan population. Am J Phys Anthropol. 2003;122:123–126. 12949832 10.1002/ajpa.10227

[42] Robinson M, Bidmos M. An assessment of the accuracy of discriminant function equations for sex determination of the femur and tibia from a South African population. Forensic Sci Int. 2011;206:212.e1–212.e5. 10.1016/j.forsciint.2010.12.009 21251773

[43] Safont S, Malgosa A, Subirà M. Sex assessment on the basis of long bone circumference. Am J Phys Anthropol. 2000;113:317–328. 11042535 10.1002/1096-8644(200011)113:3<317::AID-AJPA4>3.0.CO;2-J

[44] Slaus M, Strinovic D, Skavic J, et al. Discriminant function sexing of fragmentary and complete femora: standards for contemporary Croatia. J Forensic Sci. 2003;48:509–512. 12762518

[45] Srivastava R, Saini V, Rai R, et al. A study of sexual dimorphism in the femur among North Indians. J Forensic Sci. 2012;57:19–23. 21854380 10.1111/j.1556-4029.2011.01885.x

[46] Wysocka J, Cieślik A, Danel D. Sex estimation using measurements of the proximal femur in a historical population from Poland. Anthropological Review. 2023;86:37–49.

[47] Carvallo D, Retamal R. Sex estimation using the proximal end of the femur on a modern Chilean sample. Forensic Sci Int. 2020;2:100077.

[48] Asala S . Sex determination from the head of the femur of South African whites and blacks. Forensic Sci Int. 2001;117:15–22. 11230942 10.1016/s0379-0738(00)00444-8

[49] Asala S . The efficiency of the demarking point of the femoral head as a sex determining parameter. Forensic Sci Int. 2002;127:114–118. 12098534 10.1016/s0379-0738(02)00114-7

[50] Monum T, Prasitwattanseree S, Das S, et al. Sex estimation by femur in modern Thai population. Clin Ther. 2017;168:e203–e207. 10.7417/T.2017.2007 28612898

[51] Nwoha P . Femoral head diameters in Nigerians. Afr J Med Med Sci. 1990;19:157–161. 2120914

[52] Seidemann R, Stojanowski C, Doran G. The use of the supero-inferior femoral neck diameter as a sex assessor. Am J Phys Anthropol. 1998;107:305–313. 9821495 10.1002/(SICI)1096-8644(199811)107:3<305::AID-AJPA7>3.0.CO;2-A

[53] Stojanowski C, Seidemann R. A re-evaluation of the sex prediction accuracy of the minimum supero-inferior femoral neck diameter for modern individuals. J Forensic Sci. 1999;44:1215–1218. 10582359

[54] Alunni-Perret V, Staccini P, Quatrehomme G. Reexamination of a measurement for sexual determination using the supero-inferior femoral neck diameter in a modern European population. J Forensic Sci. 2003;48:1–4. 12762520

[55] Luna L, Bosio L, García Guraieb S, et al. Adult sex estimation from the minimum supero-inferior femoral neck diameter in a contemporary osteological sample from Buenos Aires, Argentina. Sci Justice. 2021;61:528–534. 34482932 10.1016/j.scijus.2021.06.007

[56] Ferreira MT, Vicente R, Navega D, et al. A new forensic collection housed at the University of Coimbra, Portugal: the 21st Century Identified Skeletal Collection. Forensic Sci Int. 2014;245:202.e1–202.e5. 10.1016/j.forsciint.2014.09.021 25450309

[57] Ferreira M, Coelho C, Makhoul C, et al. New data about the 21st Century Identified Skeletal Collection (University of Coimbra, Portugal). Int J Leg Med. 2020;135:1087–1094. 10.1007/s00414-020-02399-6 32857278

[58] Perini T, Oliveira G, Ornellas J, et al. Technical error of measurement in anthropometry. Revista Brasileira de Medicina Do Esporte. 2005;11:81–85.

[59] Ulijaszek S, Kerr D. Anthropometric measurement error and the assessment of nutritional status. Br J Nutr. 1999;82:165–177. 10655963 10.1017/s0007114599001348

[60] Garn S, Lewis A, Walenga A. Crown size profile pattern comparisons of 14 human populations. Arch Oral Biol. 1968;13:1235–1242. 5250248 10.1016/0003-9969(68)90079-4

[61] Koch K . Introduction to Bayesian statistics. Berlin (Germany): Springer, 2007.

[62] Irurita Olivares J, Alemán AI. Validation of the sex estimation method elaborated by Schutkowski in the Granada Osteological Collection of identified infant and young children: analysis of the controversy between the different ways of analyzing and interpreting the results. Int J Leg Med. 2016;130:1623–1632. 10.1007/s00414-016-1354-z 27002628

[63] Bocquet-Appel J . Recent advances in palaeodemography: data, techniques, patterns. London (UK): Springer; 2008.

[64] Hoppa R, Vaupel J. Paleodemography. Age distributions from skeletal samples. Cambridge (UK): Cambridge University Press; 2002.

[65] Adams B, Byrd J. Recovery, analysis, and identification of commingled human remains. New York (NY): Humana Press; 2008.

[66] Ross A, Ubelaker D, Kimmerle E. Implications of dimorphism, population variation, and secular change in estimating population affinity in the Iberian Peninsula. Forensic Sci Int. 2011;206:214.e1–214.e5. 10.1016/j.forsciint.2011.01.003 21288670

[67] DiBennardo R, Taylor J. Classification and misclassification in sexing the Black femur by discriminant function analysis. Am J Phys Anthropol. 1982;58:145–151. 7114200 10.1002/ajpa.1330580206

[68] Spradley M, Jantz R. Sex estimation in forensic anthropology: skull *versus* postcranial elements. J Forensic Sci. 2011;56:289–296. 21210801 10.1111/j.1556-4029.2010.01635.x

